# Oxygen microbubbles improve radiotherapy tumor control in a rat fibrosarcoma model – A preliminary study

**DOI:** 10.1371/journal.pone.0195667

**Published:** 2018-04-09

**Authors:** Samantha M. Fix, Virginie Papadopoulou, Hunter Velds, Sandeep K. Kasoji, Judith N. Rivera, Mark A. Borden, Sha Chang, Paul A. Dayton

**Affiliations:** 1 Eshelman School of Pharmacy, The University of North Carolina at Chapel Hill, Chapel Hill, NC, United States of America; 2 Joint Department of Biomedical Engineering, The University of North Carolina at Chapel Hill and NC State University, Chapel Hill, NC, United States of America; 3 Department of Mechanical Engineering, University of Colorado, Boulder, Colorado, United States of America; 4 Department of Radiation Oncology, The University of North Carolina at Chapel Hill, Chapel Hill, NC, United States of America; National Cancer Institute, UNITED STATES

## Abstract

Cancer affects 39.6% of Americans at some point during their lifetime. Solid tumor microenvironments are characterized by a disorganized, leaky vasculature that promotes regions of low oxygenation (hypoxia). Tumor hypoxia is a key predictor of poor treatment outcome for all radiotherapy (RT), chemotherapy and surgery procedures, and is a hallmark of metastatic potential. In particular, the radiation therapy dose needed to achieve the same tumor control probability in hypoxic tissue as in normoxic tissue can be up to 3 times higher. Even very small tumors (<2–3 mm^3^) comprise 10–30% of hypoxic regions in the form of chronic and/or transient hypoxia fluctuating over the course of seconds to days. We investigate the potential of recently developed lipid-stabilized oxygen microbubbles (OMBs) to improve the therapeutic ratio of RT. OMBs, but not nitrogen microbubbles (NMBs), are shown to significantly increase dissolved oxygen content when added to water in vitro and increase tumor oxygen levels in vivo in a rat fibrosarcoma model. Tumor control is significantly improved with OMB but not NMB intra-tumoral injections immediately prior to RT treatment and effect size is shown to depend on initial tumor volume on RT treatment day, as expected.

## Introduction

**Fig 1:**
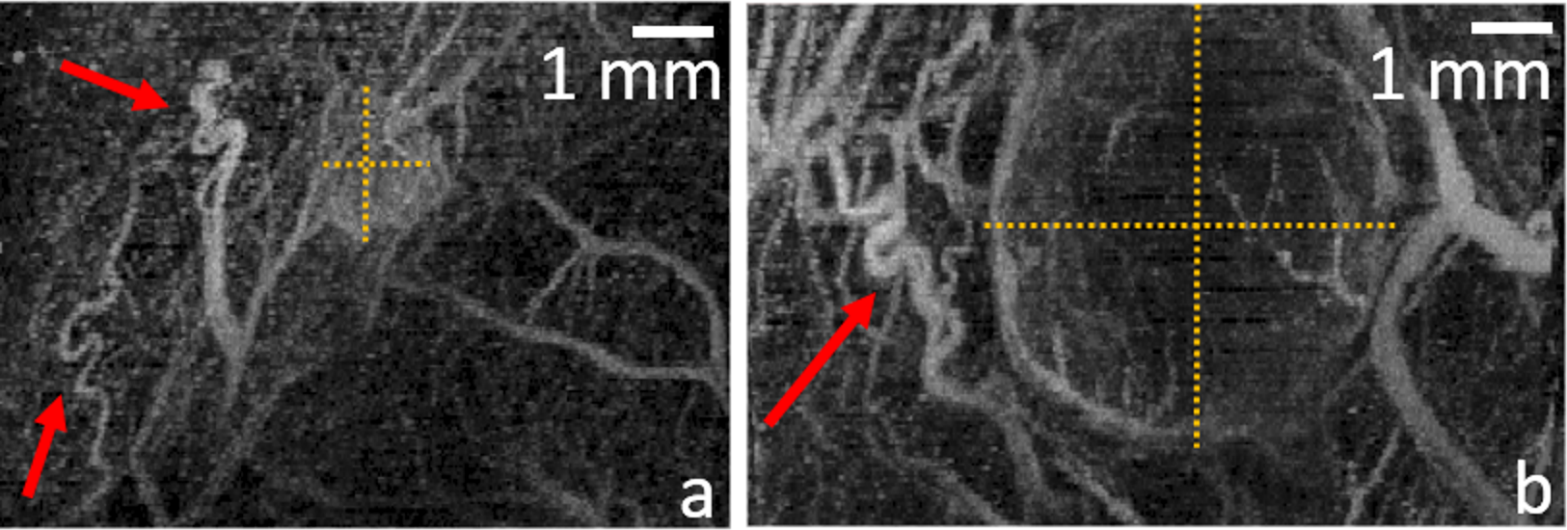
(Color online) example of acoustic angiography maximum intensity projections around tumors (tumor size denoted with dashed yellow lines) in a rat fibrosarcoma allograft, with tortuous angiogenesis extending beyond the tumor margins (red arrows). The small tumor (a) is also shown to be more enhanced, denoting its higher perfusion compared to the larger tumor (b).

Cancer affects 39.6% of Americans at some point during their lifetime [1]. Solid tumors are characterized by the presence of disorganized, tortuous, leaky vessels that promote regions of low oxygenation (hypoxia), Fig 1. Even small tumors (<2-3mm^3^) comprise 10–30% of hypoxic regions in the form of chronic and/or transient hypoxia fluctuating over the course of seconds to days [2, 3]. In fact, it has been shown repeatedly that hypoxia is a key factor in treatment failure and recurrence after treatments with radiotherapy (RT), chemotherapy and surgery [4–6]. Chronic exposure to this hypoxic environment selects for the most aggressive and resistant tumor cells, and triggers the angiogenic signaling that contributes to the overall growth of the tumor as it develops its own blood supply network. It is therefore recognized as a hallmark of metastatic potential [7–11].

Radiotherapy is one of the key primary treatment options for a variety of cancers and is used in over one million cancer patients yearly in the United States [12–14]. It is well-established that tumor hypoxia negatively impacts treatment outcome for RT [15]. In particular, the RT dose needed to achieve the same tumor control probability in hypoxic tissue as in normoxic tissue can be up to 3 times higher [4]. Hypoxia promotes radioresistance directly through the reduction of oxygen-dependent free radical damage and indirectly through biological HIF-1 complex signaling. The tumor re-oxygenation which occurs normally after RT also increases oxidative stress, leading to endothelial sensitization at the tumor level [16–18].

It is believed that transiently relieving tumor hypoxia during radiotherapy (RT) could significantly improve treatment outcome [19]. Previous in vitro studies have shown that increased oxygen presence even just a few milliseconds before or after RT significantly increases radiation-induced cancer cell damage [20]. There have been numerous previous attempts to reoxygenate tumors to this effect, including hyperbaric oxygenation, inhaled carbogen, nitroimidazoles and other radiosensitizers; however, practical administration difficulties, vasoconstriction and normal tissue toxicity have severely limited clinical translation [21–23].

Recently, the technology of oxygen microbubbles (OMB) has made several substantial advances, with the development of high-payload OMBs similarly formulated to micrometer-sized ultrasound vascular contrast agents but comprising an oxygen gas core [24]. As such, OMB have shown promise experimentally as an adjuvant cancer therapy in vivo to enhance the efficacy of oxygen-dependent therapies. In radiotherapy, they would offer the ability for localized oxygen delivery without the use of expensive dedicated equipment incompatible with radiation therapy rooms. The robust oxygen-delivery potential of OMB is demonstrated by their ability to sustain animals with otherwise fatal pneumothorax for over two hours [25], and double the survival time of asphyxiated animals [26] when delivered intra-peritoneally. In a mouse model of pancreatic cancer, OMB delivered by direct injection in the tumor have also been shown to improve the efficacy of sonodynamic therapy [27, 28]. In chemotherapy, oxygen and paclitaxel loaded microbubbles administered intravenously have shown promise as a combination therapy in an ovarian mouse xenograft model [29].

We hypothesize that these oxygen microbubbles could also be used to transiently relieve tumor hypoxia and thereby improve RT outcome if administered prior to treatment so that additional oxygen is present during radiation treatment. As a first proof of principle demonstration, in this work, we assess the potential of oxygen microbubbles to increase dissolved oxygen saturation in hypoxic solutions in vitro, increase tumor oxygenation in a rat fibrosarcoma in vivo after direct injections, and improve tumor control after RT.

## Material and methods

### Microbubble manufacturing and characterization

All glassware was cleaned with an Alconox detergent purchased from Sigma-Aldrich (St. Louis, MO) and rinsed with 18 MΩ-cm deionized water (Direct-Q, Millipore; Billerica, MA). A concentrated (10x) phosphate-buffered saline (PBS) solution from Sigma-Aldrich (St. Louis, MO) was diluted to normal concentration with deionized water and vacuum filtered through a 0.2 μm nylon membrane filter (Whatman, Kent, United Kingdom). Phospholipid 1,2-distearoyl-sn-glycero-3-phosphocholine (DSPC) was purchased from NOF (Tokyo, Japan), and polyoxyethylene-40 stearate (PEG-40S) was purchased from Sigma-Aldrich (St. Louis, MO). Oxygen and nitrogen gas were purchased from Airgas (Airgas, Radnor, PA).

DSPC and PEG-40S were weighed in dry form, mixed in a 9:1 molar ratio, and added to the filtered PBS solution to achieve a final lipid concentration of 12 mg/mL. A technique used by Feshitan et al. [25] was implemented to dissolve the DSPC and PEG-40S into the PBS solution and create a homogenous lipid solution. After adding the DSPC and PEG-40S, the mixture was heated to 65 °C and homogenized using a Branson 450 sonifier (Danbury, CT) with an output power of 25% total capability. The solution was sonicated until it appeared translucent and then stored in the refrigerator at 4 °C. Oxygen microfoam was created from a process design developed by Swanson et al. [30] to produce large volumes of oxygen microbubbles. The process developed was used to create oxygen microbubbles specifically, but the methodology is the same to produce nitrogen microbubbles with the exchange of oxygen for nitrogen gas. The process comprised an ultrasonic horn reactor enclosed in a water-cooled, continuous-flow chamber (Branson, Danburry, CT). The lipid solution was kept cool with ice packs and combined with room temperature oxygen in the reactor. The lipid solution flow rate was nearly double the flow rate of oxygen. Full sonication power was used in the reactor to emulsify the oxygen gas and the sonicated solution was collected in a cooling column to separate the oxygen microbubbles (bottom) from the macrofoam (top). The column was extracted into 60-mL syringes and centrifuged to further concentrate the oxygen microbubbles. The 60-mL syringes were placed in an Eppendorf 5804 centrifuge (Hauppauge, NY) and centrifuged at 150 relative centrifugal force (RCF) for 4 min to yield a final concentration of ~ 70 vol%. The concentrated oxygen microbubble foam was transferred into 20-mL glass serum vials (Wheaton, Millville, NJ), sealed with an oxygen headspace, and stored at 5 °C. The remaining centrifuged lipid solution was recycled and the process was repeated until the desired volume of 70 vol% oxygen microbubbles was produced. The concentration and size distribution of the oxygen microbubbles (OMBs) (n = 3 independent samples) were measured using the Coulter Counter method (Coulter Multisizer III, Beckman Coulter, Indianapolis, IN). The same methodology described above was used to make concentrated nitrogen microbubbles (NMBs) by just replacing the oxygen gas with room temperature nitrogen gas.

To extract microbubbles from a vial to be used in experiments, they were slowly pulled into a syringe through a 20-gauge needle, while a bag filled with 100% oxygen was connected to another needle in the vial top (to avoid creating a vacuum in the vial that could compromise the bubbles’ integrity, as well as minimize the introduction of room air into the vial).

### In vitro oxygen release

A fiber-optic oxygen sensor (Oxymicro, WPI, Sarasota, FL) was used to measure the dissolved oxygen content in de-ionized water before and after microbubble injection. Prior to use, the device was calibrated according to manufacturer’s instructions for a standard two-point calibration in oxygen-free water and water vapor saturated air. Calibration for automatic temperature compensation was not performed since all experiments were performed in quick succession at room temperature (centrally maintained at 22°C).

For the measurement, a beaker with 70 mL of partially degassed de-ionized water containing a magnetic stirrer was placed on a stir plate for continuous mixing and the fiber-optic measurement device recorded continuously before, during, and after OMB and NMB injections. Injections consisted of 300 μL undiluted OMB or NMB. Experiments were repeated thrice with independent vials of OMB or NMB and measurements were recorded continuously before and for at least 5 min post microbubble injection. The maximum change in dissolved oxygen saturation over the 5 min post-injection was compared between OMB and NMB groups.

### Animal model for all in vivo studies

All animal procedures were approved by the Institutional Animal Care and Use Committee of the University of North Carolina at Chapel Hill and performed in accordance with the Guide for the Care and Use of Laboratory Animals of the National Institutes of Health. Female Fisher 344 rats with subcutaneous fibrosarcoma (FSA) tumor allografts were used in all in vivo experiments. This model was chosen as the development of hypoxia in these FSA tumors has been extensively characterized [31–34]. These previous studies demonstrate hypoxia through EF5 and pimonidazole immunostaining, in addition to direct detection of pO_2_ in the tumor tissue with microelectrodes. Results demonstrate moderate hypoxia throughout much of the tumor and more severe hypoxia towards the tumor center. Briefly, tumor allografts grew after subcutaneous implantation on the right flank of 1 mm^3^ fibrosarcoma (FSA) tissue freshly resected from donor tumor-bearing rats. Animals were used for experiments 2–3 weeks after implantation, when tumors were around 1 cm in diameter. The following standardized anesthesia protocol was followed for all tumor hypoxia measurements and for radiotherapy treatment studies. Anesthesia was induced by placing the animals in an induction box for 3 min to breathe 5% vaporized isoflurane with pure oxygen as the carrier gas. Anesthesia was maintained by having the animals breathe 2.0–2.5% isoflurane with medical air as the carrier gas for the remainder of the experiment. The timing of this anesthesia protocol and the use of medical air for the primary carrier gas allowed for consistent tumor hypoxia measurements and minimized changes in blood oxygenation due to pure oxygen breathing rather than microbubble intervention. The animals’ temperatures were maintained throughout the experiments using a heated platform. When ultrasound imaging was used to evaluate tumor volume (unrelated to measuring tumor hypoxia), animals were anesthetized using vaporized isoflurane (initially 5% for induction, then 2–2.5%) with oxygen carrier gas.

### In vivo oxygen release

**Fig 2:**
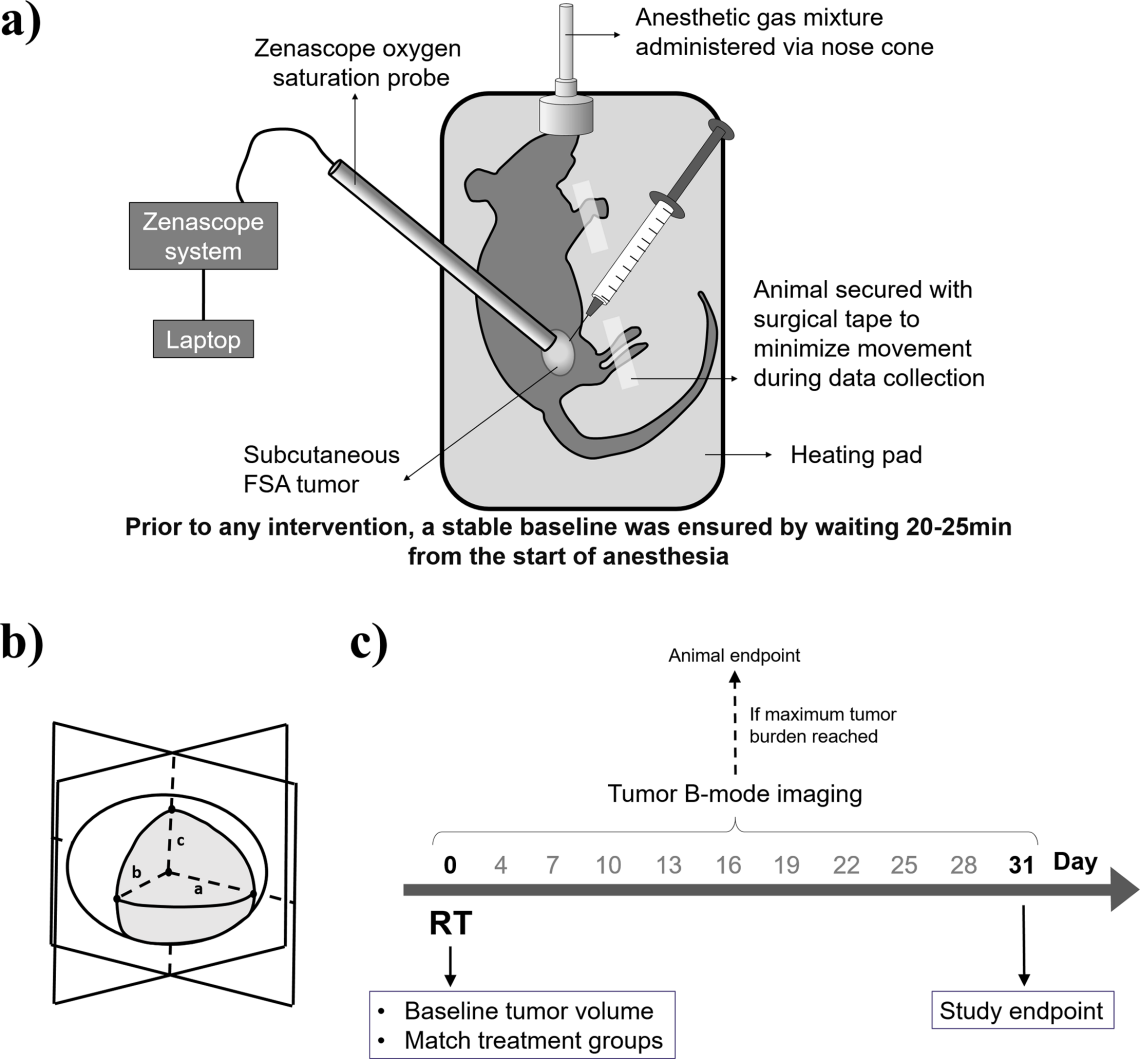
Experimental procedures. a) Schematic of the experimental set-up used for in-vivo hypoxia modulation measurements using the Zenascope system. b) Schematic of tumor volume assessment via B-mode ultrasound imaging. Two cross-sectional images were acquired and lengths a, b and c were used to calculate tumor volume. c) Radiotherapy pre and post-imaging experimental protocol.

Fisher rats with FSA tumors were anesthetized as described above, and the tumor area was shaved. Tumor oxygenation was measured continuously in real-time using a validated optical spectroscopy technique based on the absorbance of oxyhemoglobin and deoxyhemoglobin (Zenascope, Zenalux Biomedical, Durham, NC). Fig 2 shows a schematic of the experimental set-up for these measurements. Each experiment was capped at one hour from the start of anesthesia. Prior to any intervention, a stable baseline was ensured by waiting 25 min from the start of anesthesia. An oxygen challenge, defined as changing the isoflurane carrier gas from medical air to pure oxygen for 3 min, served as a positive control to ensure that an increase in blood oxygenation could be measured reliably in the tumor. Tumor hypoxia level was measured continuously before and after the following interventions:

1. OMB administration: 500 μL undiluted OMB injected intra-tumorally slowly over 30 s (n = 4);
2. Nitrogen microbubble (NMB) administration (negative control): 500 μL undiluted NMB injected intra-tumorally slowly over 30 s (n = 4).

The primary objective of this experiment was to characterize the ability of intratumoral OMB administration to reoxygenate FSA tumors, and a secondary objective was to confirm baseline hypoxia in this tumor model.

### Radiotherapy experiments

Animals were anesthetized with isoflurane and oxygen carrier gas and positioned on a heated pad, similarly to Fig 2. Two-dimensional B-mode ultrasound imaging was used to calculate tumor volume (Acuson Sequoia 512, Mountain View, CA). The largest tumor cross-sections in the sagittal and transverse planes were selected and saved after moving the transducer along these directions on a 3D motion stage. Tumor volumes were then calculated using the ellipsoid volume formula, $V=\frac{4}{3}\pi \;a\;b\;c$, where *V* is the calculated tumor volume, *a* is half the measured tumor width on the sagittal plane, *b* is half the measured tumor width on the transverse plane, and *c* is half the measured tumor depth (taking the average between the sagittal and transverse planes), Fig 2.

For a fixed dose of RT, it is well established that tumor control is strongly correlated to the initial tumor volume on the day of treatment [35–41]. Since this study aims to evaluate the feasibility of using OMB to improve radiotherapy outcome (tumor control), a matched study design with respect to initial tumor volume on the day of radiotherapy was chosen to limit the animal numbers needed. Since both the effect size of OMB and OMB dosing per tumor size are unknown prior to the study, this strategy allows to see how the results scale with respect to tumor volume and can then serve as a basis for a larger study (see Discussion for a full explanation).

For this reason, care was taken to match initial tumor sizes on the RT day between treatment groups. Tumor volume matching was achieved by implanting a few extra animals to allow selecting the closest tumor volumes possible and ordering animals into similarly sized groups after imaging, then randomizing treatment group assignment within these ordered categories. To minimize other biological variability within each experimental round, all animals were ordered on the same day (of similar age), had the same time to acclimatize to the vivarium before tumor implantation, were implanted on the same day from the same donor tumor, and were treated on the same day. Hydration and wet food packs were given to all animals irrespective of treatment (or no treatment) group. The experimental rounds resulting from this matching protocol are summarized in Table 1 and described hereafter.

**Table 1:** Experimental rounds for the radiotherapy experiments.

	RT	RT + OMB	RT + NMB	OMB alone	No treatment
ROUND 1	n = 2	n = 2	n = 2		
ROUND 2	n = 4	n = 4	n = 4	n = 4	n = 4

Animals were matched according to initial tumor volume on the day of radiotherapy, and experiments were repeated in two separate rounds: round 1 consisted of n = 2 animals per group and round 2 of n = 4 animals per group (where the experimental group conditions of ‘OMB alone’ and ‘No treatment’ were also added). Within each experimental round, the animals were the same age, had the same amount of time to acclimatize to the vivarium before tumor implantation, were implanted on the same day from the same donor tumor, and were treated on the same day.

A total of 18 animals were matched between radiotherapy treatment groups, 1) RT alone, 2) RT+OMB and 3) RT+NMB as described above, in two rounds of experiments (n = 2 per group in the first round, then n = 4 per group in the second round, total of n = 6 per group). Radiotherapy consisted of a single 15 Gy dose of 6 MV photons (2 cm x 2 cm field size) delivered using a clinical linear accelerator (Siemens Healthcare, Malvern, PA), following a previously described protocol from our group [42]. Animals were anesthetized as described above (standardized protocol with medical air as the carrier gas) and positioned on a heating pad on top of the clinical accelerator table. The skin around the tumor was gently extended and taped so that the tumor was positioned outward from the body to avoid irradiating vital organs, and a 1 cm thick tissue-mimicking bolus was placed on top of the tumor to correct for normal tissue attenuation of the radiation field meant for deeper tumors. The patient table height was adjusted using light field crosshair projected on paper prior to the start of radiation therapy. Animals in the groups receiving microbubbles were injected with 1 mL undiluted OMB or NMB intra-tumorally immediately prior to the start of RT (since we are using a clinical linear accelerator for treatment, in practice it takes 1 min to leave the treatment room and start the treatment protocol). Following RT, tumor volume was measured using B-mode ultrasound as previously described every 3 days for 31 days, as shown in Fig 2, or until the tumor reached 2.5 cm in the largest dimension, at which point animals were humanely sacrificed. Animals were sacrificed via isoflurane overdose followed by thoracotomy as a secondary means of euthanasia.

### Effect of OMB administration in the absence of radiotherapy

During the second round of radiotherapy experiments, an additional two conditions were tested: no treatment (n = 4) and OMB alone without RT (n = 4) and all animals in this round were also matched for initial tumor volume as previously described.

### Data analysis and statistical methods

All data are presented as mean ± standard deviation unless otherwise stated. Statistical significance was set a priori at p<0.05 (*). For in vitro and in vivo oxygen release measurements, the maximum difference in dissolved oxygen content after microbubble injection was compared between the nitrogen and oxygen groups using a Student’s t-test. For radiotherapy experiments, matched statistical comparison tests on the RT ‘tumor control time’, defined as the time (in days) to reach maximum tumor burden (animals below the maximum tumor burden at day 31 were included as day 32), between the treatment groups were performed: either repeated measures ANOVA with Newman-Keuls multiple comparison post-test after confirming normality, or Friedman test with Tukey’s post-test after negative normality test.

## Results

### Microbubble characterization and in vitro oxygen release

**Fig 3:**
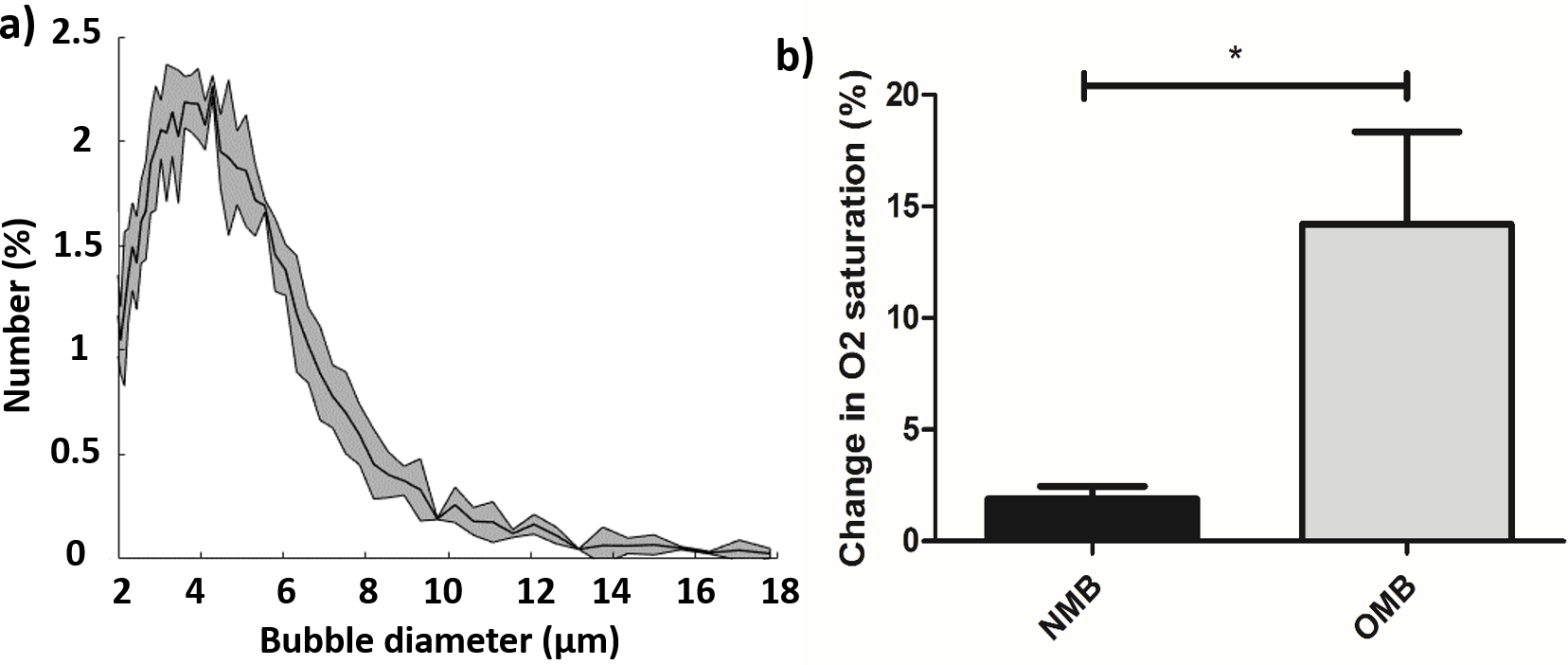
In vitro oxygen microbubble characterization. a) Measured oxygen microbubble size distribution, displayed with a diameter bin size of 0.032 μm, as mean ± standard deviation (gray area) from 3 independent samples; b) Measured change in oxygen % saturation in vitro after 300 μL OMB (n = 3) or NMB (n = 3) injection into 70 mL partially degassed water (p<0.05).

Microbubble concentration was measured as 1.3 (±0.4) × 10^9^ mL^-1^ and median bubble size was around 4 μm, as shown in Fig 3A. In vitro dissolved oxygen saturation measurements were significantly increased with the addition of 300 μL OMB into 70 mL water, by 14.2 ± 7.2%, compared to adding NMB (p = 0.04, n = 3 independent samples).

### In vivo oxygen release results

**Fig 4:**
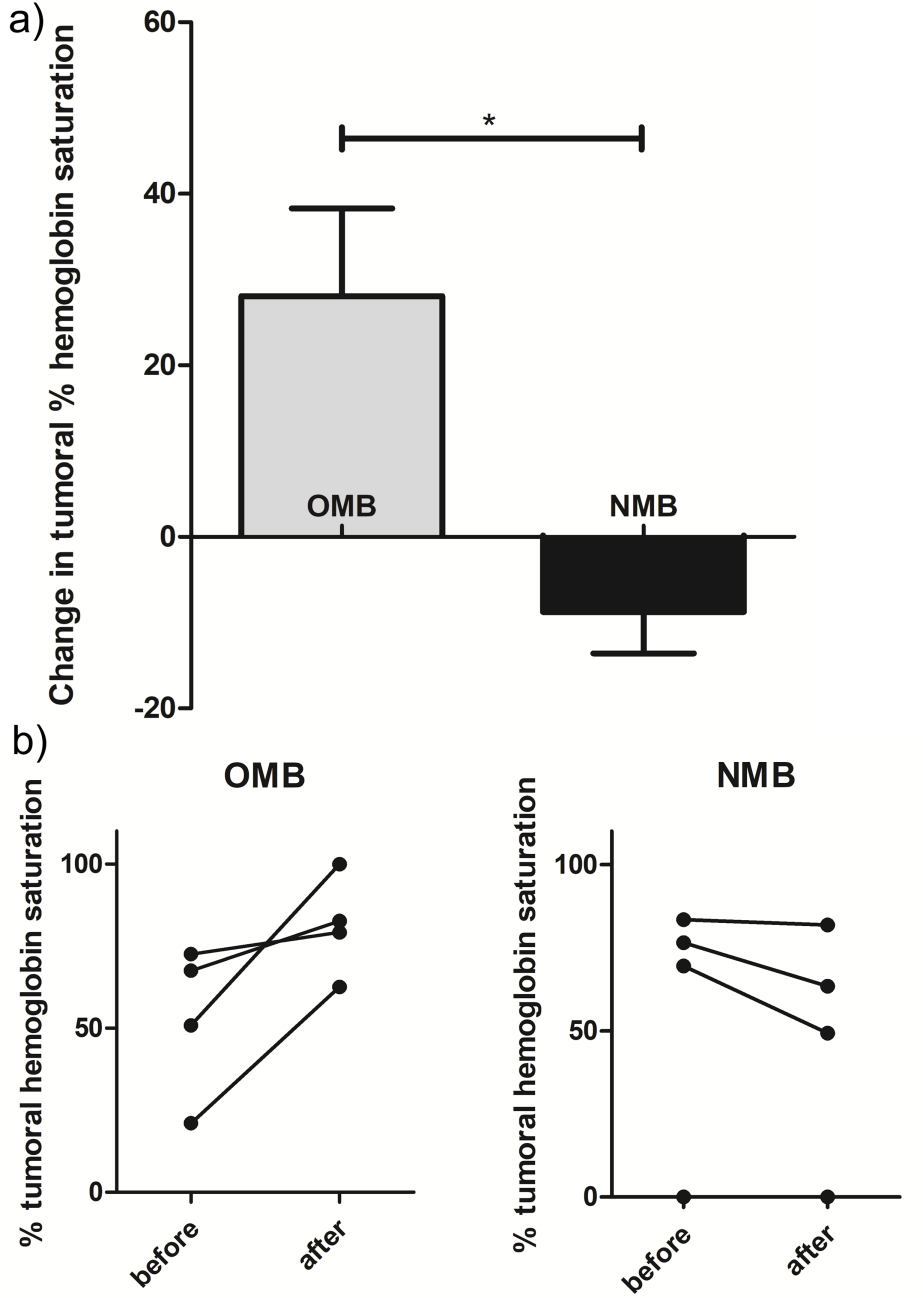
Change in tumoral oxygenation with intra-tumoral injection of OMB or NMB. The time to peak was found to be 97 s after injection on average, and the OMB-induced increase in tumoral oxygenation lasted for over 18 min on average (our protocol’s maximum 1 h experiment time meant that we could not wait for a complete return to baseline in some cases). A) Average peak change in tumoral hemoglobin saturation after OMB or NMB administration (n = 4/group). B) Individual data points showing pre- and post-injection values. This demonstrates baseline hypoxia in all tumors (0–83% hemoglobin saturation across all 8 tumors).

Consistent real-time oxygenation dynamics were recorded using a non-invasive spectroscopic measurement system by assuring prior to any intervention that sufficient time was given to achieve a stable baseline during anesthesia. OMB were shown to increase tumor oxygenation, whereas NMB lowered tumor oxygenation (Fig 4). Tumors used for this study ranged in diameter from 5mm– 17mm, approximately matching the range of tumor sizes used for subsequent radiotherapy experiments.

The average baseline percent hemoglobin saturation across all 8 tumors used for this experiment was 55 ± 30% (range from 0–83% hemoglobin saturation). These data demonstrate that the FSA model used here is indeed hypoxic. It is important to mention, however, a limitation of the Zenalux measurement system. This system measures hemoglobin saturation via optical spectroscopy. Therefore, it cannot accurately measure hypoxia deep within tissue due to the limited penetration depth of light. And thus, for large tumors we are likely measuring hemoglobin saturation only at the outer edge of the tumor rather than the center, biasing our values to be higher than what would be observed at the tumor’s center. We believe that the percent hemoglobin saturation reported here is conservative, and the tumors’ centers were likely more hypoxic than what we report.

### Radiotherapy results

Table 2 details all results from the radiotherapy experiments, for both rounds and including all controls.

**Table 2:** Individual datapoints for radiotherapy tumor control times (in days) stratified by matched initial tumor size for each treatment group, showing RT effect size depends on initial tumor volume.

	Matched initial tumor volume (cm^3^)	Tumor control (in days) for the different treatment groups					Increase in tumor control between RT and RT+OMB	
		No treatment (n = 4)	OMB alone (n = 4)	RT + NMB (n = 6)	RT (n = 6)	RT + OMB (n = 6)	In days: (RT+OMB)—RT	As percentage (%): (RT+OMB) / RT * 100–100
ROUND 2	0.1 ± 0.0	32	32	32	32	32	0	0
	0.3 ± 0.1	25	19	32	32	32	0	0
	0.6 ± 0.1	10	19	22	22^a^	31	9	41
	0.8 ± 0.1	10	10	22	22	28	6	27
ROUND 2	1.7 ± 0.2			10	16	22	6	38
	2.8 ± 0.3			7	7	10	3	43

^a^Animal died prior to experimentation end, value replaced from the NMB group since no overall difference was found between these two groups.

**Fig 5:**
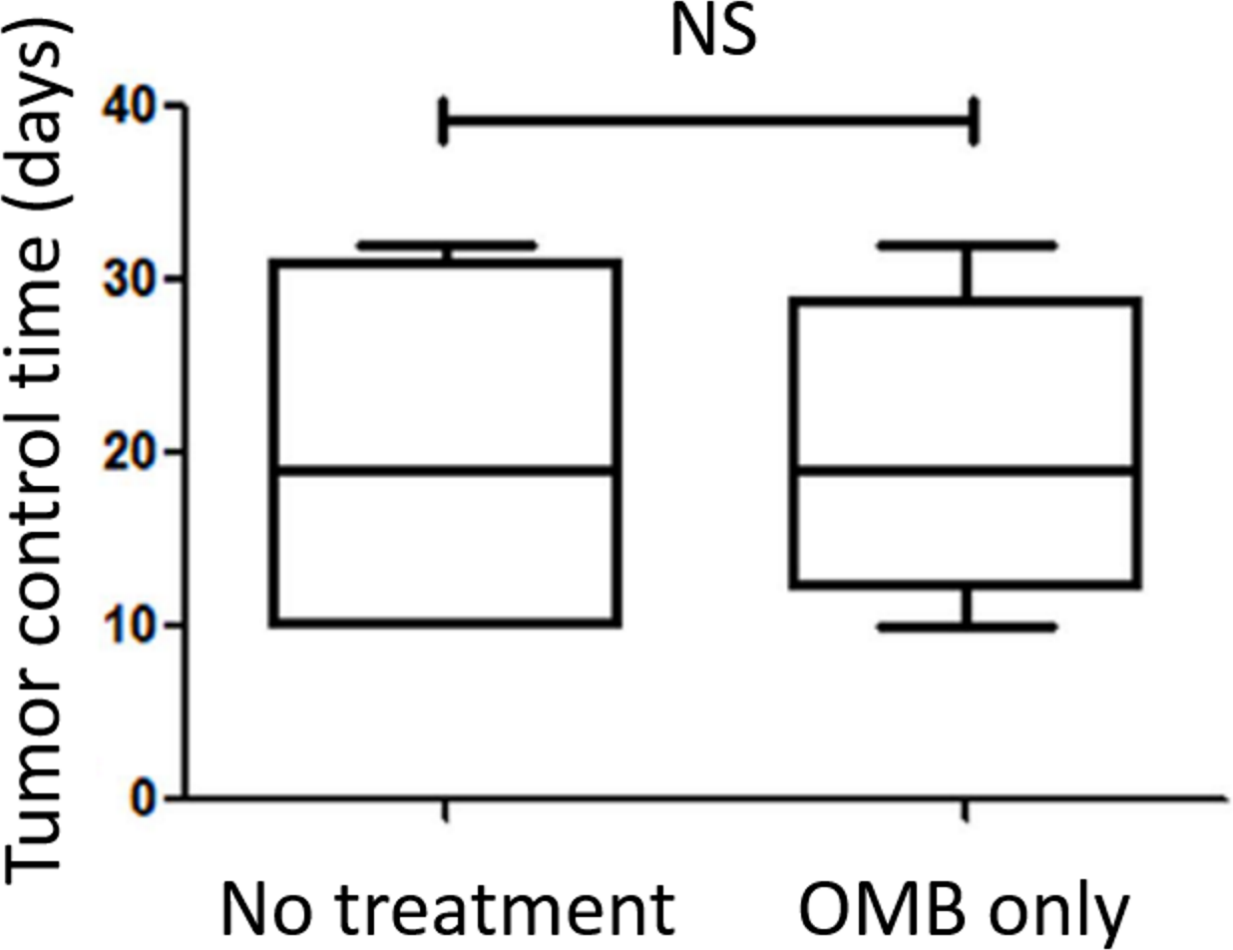
A single oxygen microbubble administration alone does not influence tumor control. No significant difference was found between the no treatment and oxygen microbubble group in the absence of any radiotherapy (n = 4 per group). Box-and-whisker plots represent all data from the No treatment and OMB alone controls.

**Fig 6:**
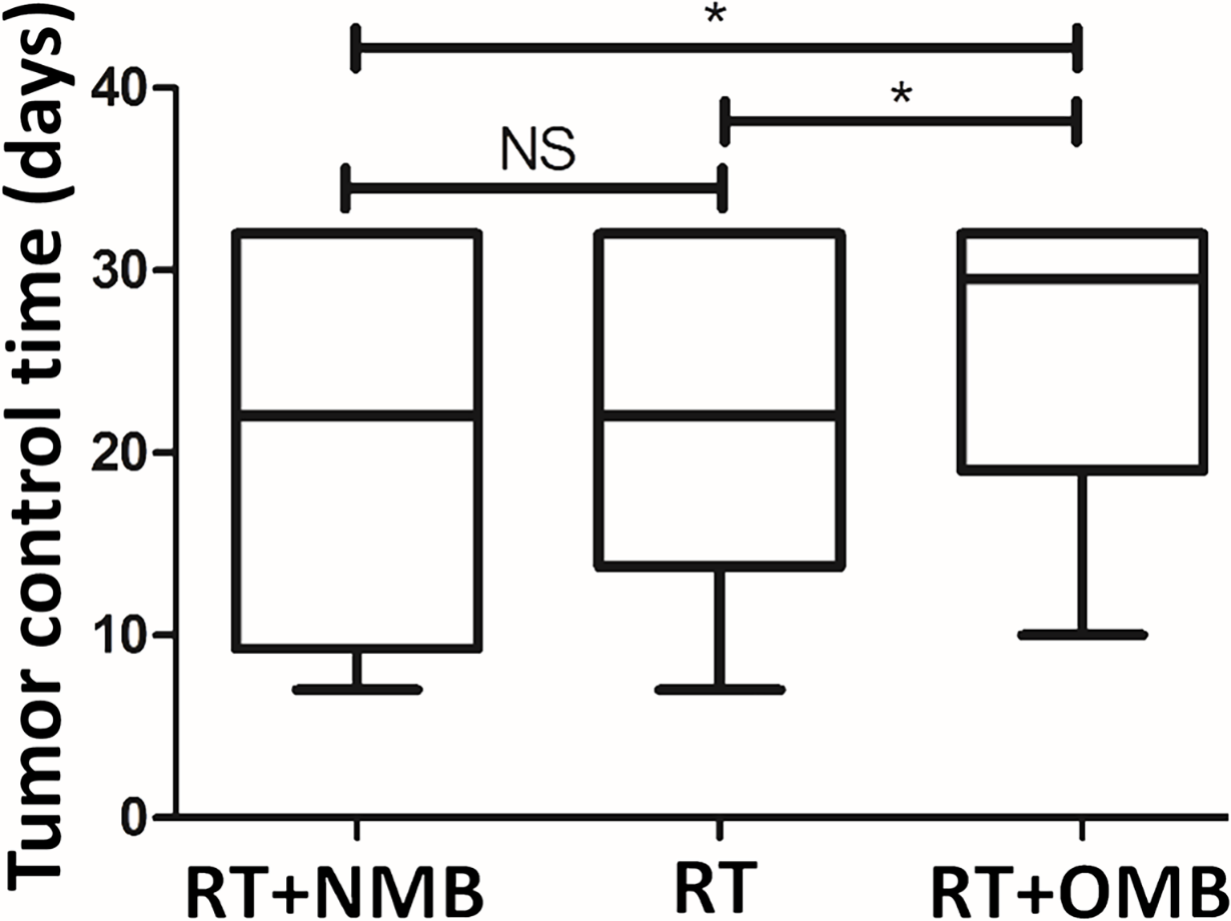
Tumor control time comparison between RT groups. OMB significantly improve RT outcome, whereas NMB as controls do not (n = 6 per group). Box-and-whisker plots show all data from the three radiotherapy treatment groups.

In Round 2, we included two additional control groups (No treatment and OMB alone, n = 4/group) to ensure that the OMB administration did not influence tumor growth in the absence of RT. Indeed, no significant difference was shown in tumor control between the animals receiving no treatment and those having received a single OMB administration in the absence of any radiation treatment, as shown in Fig 5. Note: these controls were not included in Round 1 of this study. Therefore, the number of animals and range of tumor sizes tested for these control groups did not match the entire range of tumor sizes used for the radiation treatment groups (RT, RT+NMB, and RT+OMB). As such, we have not drawn direct comparisons between tumor control time of the No treatment and OMB only groups (Fig 5) with those of the three radiation treatment groups (Fig 6).

From the animals receiving RT, 17/18 successfully completed the study (one of the rats in the RT alone group died during anesthesia prior to the completion of the RT treatment protocol and was therefore excluded from the analysis). Initial RT results show that intra-tumoral OMB improve tumor control after radiotherapy (p<0.05, n = 6 per group), Fig 6, and that initial tumor size significantly affects RT outcome, Table 2.

## Discussion

### OMB modulate tumor hypoxia

In this study, we demonstrate the oxygen payload of OMB in vitro and in vivo, before showing that they can be used to significantly improve radiotherapy tumor control in a fibrosarcoma model in vivo. In vitro, the addition of OMB to hypoxic solution increases the amount of dissolved oxygen, as expected, and has significantly higher effect than that of the NMB control. The very slight increase shown with NMB is due to the fact that these microbubbles were added to hypoxic solutions and measurements were collected over a 5 min period, so the liquid uptakes oxygen molecules through its surface area in contact with ambient air over this time (gas exchange towards equilibrium). In vivo, direct OMB injections into fibrosarcoma tumors are shown to significantly increase tumoral oxygenation, whereas the NMB injection control had the opposite effect. This increase is very fast (peaks around 90 s post injection) and the remains elevated for over 15 min, which is consistent with the improved tumor control observed when combining RT with OMB administration in vivo.

### Radiotherapy improvement dependency on initial tumor volume

**Fig 7:**
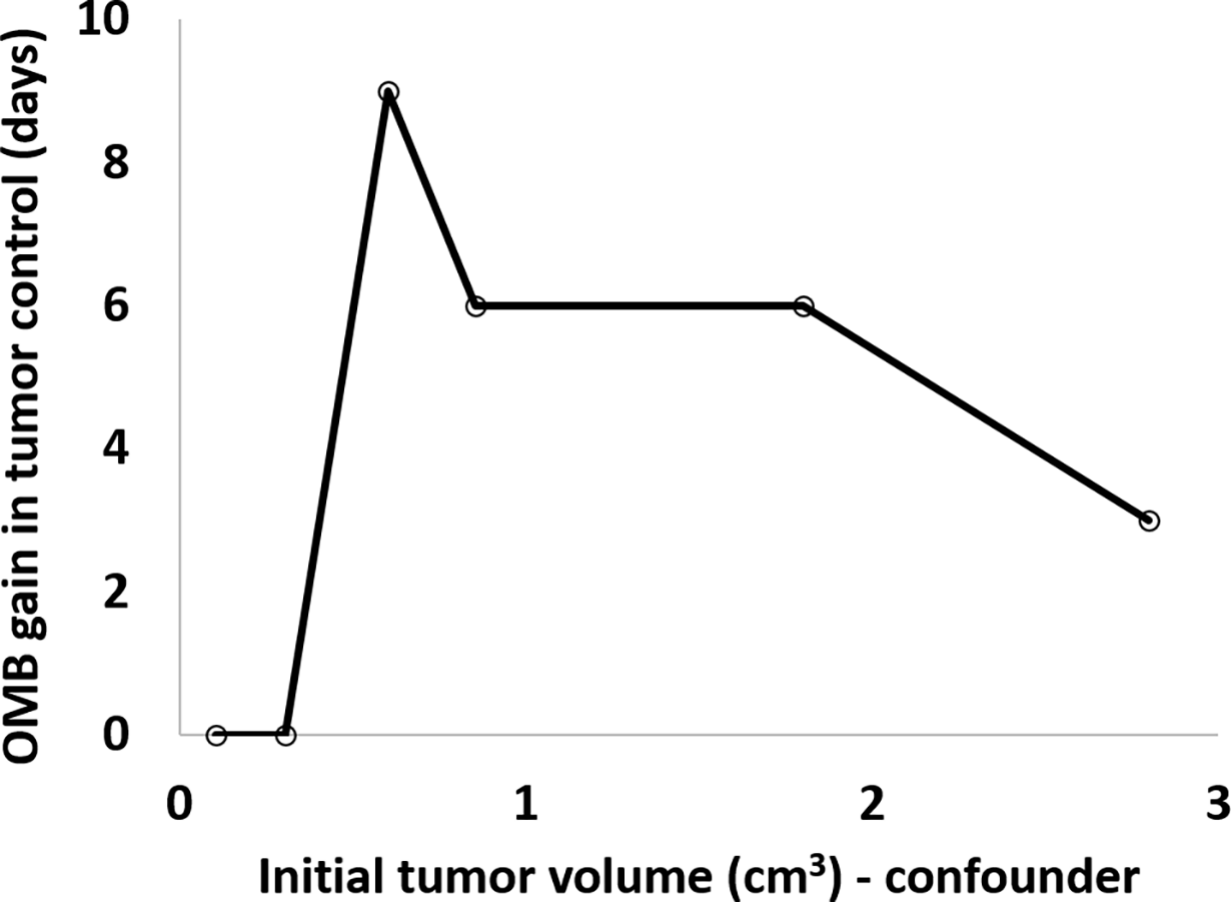
Plot of gain in tumor control time against initial tumor volume, the confounding variable. An on/off effect (threshold) is observed around 0.5 cm^3^ initial tumor volume. Below this size, tumors are controlled for 31 days with RT alone. Above this size, tumors are large enough that RT alone cannot control them for 31 days, and therefore, OMB administration can provide a substantial improvement in tumor control time. Additionally, the benefit offered by OMB administration diminishes as initial tumor volume exceeds ~2 cm^3^. Given our study design, the slope describing the inverse relationship between improvement in tumor control and initial tumor volume (above the threshold value of 0.5 cm^3^) could in theory guide optimal OMB dosing with respect to tumor volume (confounder) in order to maximize tumor control benefit for a given RT dose.

The results show that for a fixed RT dose and fixed OMB dose, the gain in tumor control time depends on initial tumor volume, Table 2. As expected, we found that OMB administration offered the greatest benefit for intermediately sized tumors (initial volume 0.6–1.7 cm^3^). Over this size range, we observed survival benefits of 6–9 days (OMB+RT vs. RT alone), Table 2 and Fig 7. Conversely, for very small tumors (<0.5 cm^3^ initial volume), the tumors likely have not yet developed extensive hypoxia, and the radiotherapy dose is already very efficient for tumor control. Thus, OMB do not significantly improve the RT efficacy for small tumors (Fig 7). This observation is also biased by the fact that our observation time was capped at 31 days, resulting in right censoring of all tumors that were still controlled by that time. For very large tumors (>2 cm^3^ initial volume), the absolute gain in tumor control (in days) drops (Fig 7). Since these are likely to be very hypoxic, and we are always injecting the same OMB dose, this is probably not enough to reoxygenate these large tumors efficiently, thus limiting therapeutic gain. Interestingly, when comparing the increase in tumor control offered by OMB administration as a percentage of that offered by RT alone, we find fairly consistent improvement of ~35% for all tumors larger than a threshold initial volume of 0.5 cm^3^ (Table 2, rightmost column).

With further optimization, we do believe that OMB administration holds the potential to offer meaningful improvements in RT-mediated tumor control over a wide range of tumor volumes. Here, OMB did not improve RT outcomes for those rats with small tumors simply because the RT dose administered was already sufficient to provide a near complete response. We hypothesize that if the RT dose was reduced for this cohort of animals, we would still be able to achieve complete tumor regression through the administration of OMB. This is attractive since lowering the RT dose would reduce exposure of healthy tissue to radiation and limit associated side effects. Similarly, we believe that we could achieve more substantial control of large tumors by either increasing the OMB dose, RT dose, or both.

### Limitations and future work

A limitation of this study is the relatively small sample size used in the assessment of OMB administration for improved RT. Nevertheless, our results are consistent between two completely independent rounds of experiments, and the benefit offered by OMB administration is large enough to be statistically significant despite the relatively small sample size. It is reasonable to assume that optimization of the dosages and administration can result in even greater improvement in tumor control. Now that a first demonstration has been established including a comparison to NMB administration, a future study could concentrate on establishing the radiotherapy dose-modifying factor (DMF) resulting from OMB (i.e. the reduction in RT dose required to achieve the same tumor control probability when OMB are administered as an adjunct therapy). To do so, animals are randomized between the RT alone and RT+OMB groups, and the RT dose necessary to control 50% of tumors is calculated from logistical regression for each group; DMF is then calculated as the ratio between these RT doses. Nevertheless, we estimate that a comprehensive assessment like the one described above requires n = 100 animals from the preliminary data presented here, and thus is not warranted until further OMB administration optimization is undertaken (see next Discussion section *Potential for clinical translation*).

Throughout this study we used a rat FSA tumor model previously characterized to develop hypoxic tumors. We confirmed baseline hypoxia via spectroscopic measurement of hemoglobin saturation. However, a limitation is that we did not confirm tumor hypoxia with a second method (e.g. immunostaining of key hypoxia markers). In future work, we plan to characterize tumor-size-dependent hypoxia and the effect of OMB administration on hypoxia in more detail using histology. We are also interested in studying in more detail the effect of tumor volume on the efficiency of OMB-mediated reoxygenation.

### Potential for clinical translation

Despite considerable progress in early detection and treatment options in multiple cancers over the last decade, cancer remains difficult to treat in advanced disease stages and radioresistance and recurrence at the primary tumor site remain significant clinical challenges.

#### Direct tumoral injections

A number of solid tumors are accessible for direct injections clinically. In particular, head and neck cancer treated with external beam radiation therapy are particularly hypoxic [43, 44] yet shallow enough for direct injections. Furthermore, more deeply seated tumors such as those of the pancreas, liver, or colon can be accessed for intratumoral injection with ultrasound, endoscopic ultrasound or computed tomography image guidance [45]. Additionally, a direct access for OMB with RT can be found in brachytherapy (clinically approved) which uses guiding tubes to feed radiation sources inside solid tumors where they irradiate for a few seconds before being retracted or are left implanted for lower irradiation over time [46]. Therefore, this direct tumoral injection of OMB could potentially be clinically translatable in the long term through brachytherapy co-administration (through one of the guiding tubes) or some needle accessible solid tumors with external beam radiation.

In human clinical studies, intratumoral injections of 20–40% of the total tumor volume have been reported [45, 47]. Here, we provided a consistent intratumoral OMB dose of 0.5 mL, regardless of tumor volume. It is promising that we found substantial survival benefit for intermediately sized tumors (e.g. 1.7 cm^3^), where the OMB injection volume corresponded to 28% of the tumor volume and was therefore within the clinically achievable range. Future efforts will be aimed at optimizing OMB dose with respect to baseline tumor hypoxia and tumor volume, after which we anticipate being able to achieve substantial reoxygenation with relevant OMB dose volumes across a wide range of tumor sizes.

#### Intravenous administration

Clinically, fractionated dose RT treatment plans, where smaller doses of radiation are administered repeatedly typically five days a week over the course of several weeks or months, were developed to spare healthy tissue toxicity, taking advantage of the better repair capability of healthy tissue compared to tumor cells. Oxygen microbubbles are similarly formulated to microbubble ultrasound contrast agents used for imaging, but comprise an oxygen gas core instead of heavy molecular weight gases. Due to their micrometer size scale, similar to that of a red blood cell, microbubble contrast agents are confined to the vascular space after being intravenously administrated and serve as an ultrasound blood pool marker. Depending on the pressure of the incident ultrasound wave, microbubbles will respond by either stably oscillating or by bursting.

Therefore, local release of oxygen from OMB following intravenous administration could be achieved using focused ultrasound in the tumor region. This would be minimally invasive and greatly advantageous in the context of fractionated dose RT. Such local reoxygenation may allow for similar tumor control with even smaller radiation doses or fewer total treatments. We have previously demonstrated in vitro that ultrasound application significantly enhances oxygen delivery from OMB [48].

In addition to the potential use of ultrasound for image-guided locally triggered oxygen release in the tumor, its ability to make microbubbles oscillate also offers a useful therapeutic target in relation to RT. Indeed, it has been shown with (non-oxygen) microbubbles that stable cavitation oscillation under ultrasound in the vasculature of the tumor prior to RT increases radiation damage to these tumor vessels in a mouse model of prostate cancer treated with radiotherapy [49]. In addition to inducing tumor cell death, RT also damages the endothelium of tumor vasculature, leading to additional tumor damage as it loses its blood supply network post-treatment [50, 51]. As such, endothelial sensitization using acoustically active agents such as microbubbles could offer an additional therapeutic target, as they mechanically oscillate near vessel boundaries under appropriate ultrasound conditions. Importantly, this promising result was achieved with non-oxygen microbubbles, so we anticipate that the additional target of hypoxia modulation would further improve these results as they target a complementary, radiosensitizing pathway.

Nevertheless, intravenous administration requires OMB that are stable enough to reach the tumor and retain their oxygen gas before being disrupted by ultrasound locally. In principle, the oxygen payload of OMBs would be retained for a longer duration in circulation if the carrier gas is pure oxygen rather than air. Additionally, the OMB formulation used in this work was designed to achieve rapid oxygen release for peritoneal microbubble oxygenation [25, 26]. The OMBs could be reformulated to increase circulation persistence and oxygen payload delivery to the tumor vasculature following intravenous administration.

#### Particular impact in RT

Finally, two specific radiotherapy targets merit further mention with respect to OMB hypoxia modulation.

First, stereotactic radiosurgery (SRS) and stereotactic body radiotherapy (SBRT) involve delivery of one or a few large dose fractions (e.g., 8–20 Gy) to the tumor volume. This approach has shown particularly promising results for inoperable early stage tumors (e.g., lung and prostate cancers) that are small, while sparing surrounding normal tissue from irradiation. However, many tumors are hypoxic and thus radio-resistant. The SRS/SBRT procedures use only a few fractions and cannot take advantage of radiotherapy-induced tumoral re-oxygenation as the conventionally fractionated RT (with 30 daily fractions) can. For fractionated RT, the surviving hypoxic cancer cells after one irradiation dose are re-oxygenated and so less hypoxic at the time of the next dose [52]. Since this is not the case for SRS and SBRT, hypoxia is deemed an even more important adjuvant therapeutic target for these treatments [53].

Secondly, 40% of patients are anemic prior to receiving RT and RT also often induces anemia [54–56]. This has important implications for tumoral hypoxia, since the decreased ability of blood to carry oxygen will also make the tumor resistant to radiation damages. It has been demonstrated that anemia is associated with lower RT local tumor control in head and neck cancers [57]. As such, an oxygen delivery system that does not rely on red blood cells such as oxygen microbubbles could significantly benefit this patient subpopulation in particular [58, 59].

## Conclusions

In conclusion, our data show that oxygen microbubbles administered by direct intra-tumoral injection in fibrosarcoma allografts in vivo are capable of increasing tumoral oxygenation significantly for tens of minutes, whereas control nitrogen microbubble injection reduces tumoral oxygenation. Furthermore, a preliminary study with a fixed microbubble dose and radiotherapy protocol shows that oxygen microbubbles significantly improve radiotherapy tumor control. This constitutes the first demonstration that OMB can improve RT outcome. The tumor control time improvement is heavily dependent on the initial tumor volume as expected for any fixed dose RT study. Smaller tumors are expected to be less hypoxic and easier to control to the end of our predetermined study observation period with radiotherapy alone, whereas large tumors are likely more hypoxic.

The ability to measure the real-time dynamics of OMB-induced tumor hypoxia modulation could also be used to inform other tumor re-oxygenation adjuvant therapies, as well as optimize the dose and timings for RT. As such, future studies will concentrate on investigating administration routes and dosages. In particular, the ability to administer OMB intravenously remains most attractive due to being minimally invasive and potentially allowing for an image-guided, ultrasound-triggered release mechanism locally. This in turn offers the largest clinical translation applicability with repeated fractionated dose radiotherapy protocols and could harness endothelial sensitization as an additional therapeutic-enhancing mechanism.

## Supporting information

S1 Tables Tables of raw data. (DOCX)
