# Supplementary material for: Oxygen microbubbles improve radiotherapy tumor control in a rat fibrosarcoma model – A preliminary study
Source: PLoS One. 2018 Apr 9;13(4):e0195667. doi: 10.1371/journal.pone.0195667 (PMC5891067; doi:10.1371/journal.pone.0195667)
Supplement: S1 Tables — (DOCX) [file pone.0195667.s001.DOCX]

**Supplementary Table S1.** Measured oxygen microbubble size distribution from 3 independent samples

| **Bubble diameter bin** | **Number (%) Sample 1** | **Number (%) Sample 2** | **Number (%) Sample 3** |
| --- | --- | --- | --- |
| 0.71 | 0.772889 | 0.93149 | 1.16059 |
| 0.717692 | 0.939358 | 0.85637 | 0.947418 |
| 0.725468 | 0.97503 | 0.871394 | 0.947418 |
| 0.733328 | 0.844233 | 0.796274 | 0.852676 |
| 0.741274 | 1.08205 | 0.811298 | 1.01847 |
| 0.749305 | 0.689655 | 0.70613 | 0.947418 |
| 0.757423 | 0.713436 | 0.615986 | 0.828991 |
| 0.76563 | 0.749108 | 0.811298 | 0.900047 |
| 0.773925 | 0.760999 | 0.70613 | 0.828991 |
| 0.78231 | 0.820452 | 0.886418 | 1.01847 |
| 0.790786 | 0.570749 | 0.841346 | 0.828991 |
| 0.799353 | 0.665874 | 0.85637 | 0.78162 |
| 0.808014 | 0.689655 | 0.691106 | 0.805306 |
| 0.816768 | 0.78478 | 0.93149 | 0.710564 |
| 0.825618 | 0.689655 | 0.796274 | 0.757935 |
| 0.834563 | 0.546968 | 0.661058 | 0.592136 |
| 0.843605 | 0.606421 | 0.736178 | 0.686878 |
| 0.852745 | 0.677765 | 0.676082 | 0.686878 |
| 0.861984 | 0.642093 | 0.495793 | 0.757935 |
| 0.871323 | 0.749108 | 0.510817 | 0.78162 |
| 0.880763 | 0.546968 | 0.615986 | 0.663193 |
| 0.890306 | 0.677765 | 0.646034 | 0.663193 |
| 0.899952 | 0.677765 | 0.420673 | 0.473709 |
| 0.909703 | 0.487515 | 0.570913 | 0.757935 |
| 0.919559 | 0.511296 | 0.63101 | 0.544766 |
| 0.929522 | 0.439952 | 0.450721 | 0.592136 |
| 0.939593 | 0.653983 | 0.63101 | 0.947418 |
| 0.949773 | 0.570749 | 0.465745 | 0.52108 |
| 0.960063 | 0.749108 | 0.676082 | 0.544766 |
| 0.970465 | 0.618312 | 0.465745 | 0.710564 |
| 0.980979 | 0.59453 | 0.646034 | 0.639507 |
| 0.991607 | 0.380499 | 0.525841 | 0.544766 |
| 1.00235 | 0.487515 | 0.420673 | 0.544766 |
| 1.01321 | 0.546968 | 0.676082 | 0.639507 |
| 1.02419 | 0.523187 | 0.480769 | 0.592136 |
| 1.03529 | 0.475624 | 0.375601 | 0.544766 |
| 1.0465 | 0.499405 | 0.480769 | 0.663193 |
| 1.05784 | 0.58264 | 0.345553 | 0.52108 |
| 1.0693 | 0.59453 | 0.435697 | 0.402653 |
| 1.08089 | 0.404281 | 0.555889 | 0.402653 |
| 1.0926 | 0.475624 | 0.525841 | 0.307911 |
| 1.10444 | 0.428062 | 0.390625 | 0.497395 |
| 1.1164 | 0.380499 | 0.450721 | 0.568451 |
| 1.1285 | 0.344828 | 0.405649 | 0.355282 |
| 1.14072 | 0.463734 | 0.570913 | 0.497395 |
| 1.15308 | 0.39239 | 0.480769 | 0.402653 |
| 1.16558 | 0.416171 | 0.300481 | 0.355282 |
| 1.1782 | 0.428062 | 0.585938 | 0.284225 |
| 1.19097 | 0.297265 | 0.375601 | 0.378967 |
| 1.20387 | 0.439952 | 0.330529 | 0.331596 |
| 1.21692 | 0.428062 | 0.315505 | 0.284225 |
| 1.2301 | 0.344828 | 0.465745 | 0.331596 |
| 1.24343 | 0.321046 | 0.300481 | 0.331596 |
| 1.2569 | 0.344828 | 0.390625 | 0.402653 |
| 1.27052 | 0.344828 | 0.420673 | 0.426338 |
| 1.28428 | 0.451843 | 0.360577 | 0.450024 |
| 1.2982 | 0.332937 | 0.330529 | 0.402653 |
| 1.31226 | 0.368609 | 0.450721 | 0.378967 |
| 1.32648 | 0.356718 | 0.240385 | 0.378967 |
| 1.34085 | 0.309156 | 0.360577 | 0.236855 |
| 1.35538 | 0.451843 | 0.330529 | 0.355282 |
| 1.37007 | 0.273484 | 0.345553 | 0.284225 |
| 1.38491 | 0.309156 | 0.255409 | 0.307911 |
| 1.39991 | 0.356718 | 0.240385 | 0.331596 |
| 1.41508 | 0.356718 | 0.270433 | 0.26054 |
| 1.43041 | 0.285375 | 0.315505 | 0.26054 |
| 1.44591 | 0.20214 | 0.270433 | 0.189484 |
| 1.46158 | 0.297265 | 0.285457 | 0.26054 |
| 1.47741 | 0.356718 | 0.270433 | 0.307911 |
| 1.49342 | 0.39239 | 0.270433 | 0.307911 |
| 1.5096 | 0.285375 | 0.405649 | 0.426338 |
| 1.52595 | 0.321046 | 0.240385 | 0.355282 |
| 1.54249 | 0.297265 | 0.345553 | 0.142113 |
| 1.5592 | 0.309156 | 0.300481 | 0.450024 |
| 1.57609 | 0.332937 | 0.345553 | 0.26054 |
| 1.59317 | 0.214031 | 0.435697 | 0.213169 |
| 1.61043 | 0.356718 | 0.240385 | 0.355282 |
| 1.62788 | 0.285375 | 0.240385 | 0.142113 |
| 1.64552 | 0.225922 | 0.270433 | 0.331596 |
| 1.66334 | 0.285375 | 0.120192 | 0.26054 |
| 1.68137 | 0.225922 | 0.285457 | 0.331596 |
| 1.69958 | 0.249703 | 0.405649 | 0.450024 |
| 1.718 | 0.332937 | 0.330529 | 0.165798 |
| 1.73661 | 0.225922 | 0.270433 | 0.284225 |
| 1.75543 | 0.225922 | 0.240385 | 0.307911 |
| 1.77444 | 0.285375 | 0.120192 | 0.26054 |
| 1.79367 | 0.380499 | 0.210337 | 0.26054 |
| 1.8131 | 0.273484 | 0.390625 | 0.213169 |
| 1.83275 | 0.309156 | 0.315505 | 0.189484 |
| 1.8526 | 0.273484 | 0.345553 | 0.142113 |
| 1.87268 | 0.237812 | 0.240385 | 0.307911 |
| 1.89297 | 0.332937 | 0.210337 | 0.284225 |
| 1.91347 | 0.237812 | 0.330529 | 0.236855 |
| 1.93421 | 0.344828 | 0.375601 | 0.213169 |
| 1.95516 | 0.309156 | 0.405649 | 0.307911 |
| 1.97635 | 0.285375 | 0.315505 | 0.26054 |
| 1.99776 | 0.309156 | 0.15024 | 0.26054 |
| 2.0194 | 0.225922 | 0.285457 | 0.165798 |
| 2.04128 | 0.166468 | 0.465745 | 0.284225 |
| 2.0634 | 0.321046 | 0.315505 | 0.189484 |
| 2.08575 | 0.404281 | 0.360577 | 0.165798 |
| 2.10835 | 0.356718 | 0.270433 | 0.142113 |
| 2.13119 | 0.356718 | 0.435697 | 0.26054 |
| 2.15429 | 0.368609 | 0.255409 | 0.213169 |
| 2.17763 | 0.297265 | 0.300481 | 0.378967 |
| 2.20122 | 0.249703 | 0.330529 | 0.331596 |
| 2.22507 | 0.428062 | 0.420673 | 0.165798 |
| 2.24918 | 0.439952 | 0.495793 | 0.236855 |
| 2.27354 | 0.344828 | 0.360577 | 0.26054 |
| 2.29818 | 0.356718 | 0.465745 | 0.307911 |
| 2.32308 | 0.439952 | 0.555889 | 0.402653 |
| 2.34825 | 0.368609 | 0.315505 | 0.307911 |
| 2.37369 | 0.39239 | 0.360577 | 0.331596 |
| 2.39941 | 0.404281 | 0.360577 | 0.378967 |
| 2.4254 | 0.428062 | 0.330529 | 0.307911 |
| 2.45168 | 0.344828 | 0.465745 | 0.142113 |
| 2.47824 | 0.356718 | 0.405649 | 0.378967 |
| 2.50509 | 0.451843 | 0.465745 | 0.165798 |
| 2.53223 | 0.428062 | 0.375601 | 0.473709 |
| 2.55967 | 0.368609 | 0.570913 | 0.402653 |
| 2.5874 | 0.380499 | 0.405649 | 0.307911 |
| 2.61544 | 0.499405 | 0.405649 | 0.497395 |
| 2.64377 | 0.487515 | 0.600962 | 0.284225 |
| 2.67242 | 0.416171 | 0.405649 | 0.307911 |
| 2.70137 | 0.535077 | 0.585938 | 0.497395 |
| 2.73064 | 0.487515 | 0.585938 | 0.473709 |
| 2.76022 | 0.499405 | 0.390625 | 0.307911 |
| 2.79013 | 0.368609 | 0.570913 | 0.378967 |
| 2.82036 | 0.487515 | 0.480769 | 0.307911 |
| 2.85092 | 0.439952 | 0.676082 | 0.236855 |
| 2.8818 | 0.535077 | 0.495793 | 0.497395 |
| 2.91303 | 0.59453 | 0.555889 | 0.592136 |
| 2.94459 | 0.523187 | 0.480769 | 0.615822 |
| 2.97649 | 0.546968 | 0.525841 | 0.402653 |
| 3.00874 | 0.463734 | 0.360577 | 0.52108 |
| 3.04134 | 0.606421 | 0.525841 | 0.592136 |
| 3.07429 | 0.499405 | 0.495793 | 0.402653 |
| 3.1076 | 0.630202 | 0.495793 | 0.355282 |
| 3.14127 | 0.59453 | 0.540865 | 0.473709 |
| 3.1753 | 0.606421 | 0.570913 | 0.450024 |
| 3.2097 | 0.558859 | 0.585938 | 0.450024 |
| 3.24448 | 0.511296 | 0.70613 | 0.52108 |
| 3.27963 | 0.701546 | 0.480769 | 0.473709 |
| 3.31516 | 0.487515 | 0.495793 | 0.450024 |
| 3.35108 | 0.428062 | 0.615986 | 0.284225 |
| 3.38739 | 0.523187 | 0.585938 | 0.639507 |
| 3.42409 | 0.713436 | 0.465745 | 0.355282 |
| 3.46119 | 0.487515 | 0.585938 | 0.378967 |
| 3.49869 | 0.653983 | 0.480769 | 0.639507 |
| 3.5366 | 0.546968 | 0.450721 | 0.378967 |
| 3.57491 | 0.499405 | 0.661058 | 0.52108 |
| 3.61364 | 0.606421 | 0.600962 | 0.52108 |
| 3.6528 | 0.653983 | 0.585938 | 0.497395 |
| 3.69237 | 0.523187 | 0.676082 | 0.544766 |
| 3.73238 | 0.58264 | 0.615986 | 0.450024 |
| 3.77282 | 0.39239 | 0.450721 | 0.568451 |
| 3.81369 | 0.570749 | 0.600962 | 0.473709 |
| 3.85501 | 0.58264 | 0.540865 | 0.473709 |
| 3.89678 | 0.665874 | 0.495793 | 0.473709 |
| 3.939 | 0.546968 | 0.495793 | 0.615822 |
| 3.98168 | 0.451843 | 0.585938 | 0.497395 |
| 4.02482 | 0.665874 | 0.495793 | 0.426338 |
| 4.06842 | 0.546968 | 0.450721 | 0.402653 |
| 4.1125 | 0.499405 | 0.585938 | 0.615822 |
| 4.15706 | 0.535077 | 0.420673 | 0.544766 |
| 4.2021 | 0.58264 | 0.585938 | 0.568451 |
| 4.24762 | 0.558859 | 0.525841 | 0.544766 |
| 4.29365 | 0.570749 | 0.691106 | 0.663193 |
| 4.34017 | 0.653983 | 0.691106 | 0.497395 |
| 4.38719 | 0.451843 | 0.435697 | 0.52108 |
| 4.43472 | 0.451843 | 0.600962 | 0.284225 |
| 4.48277 | 0.451843 | 0.360577 | 0.450024 |
| 4.53134 | 0.558859 | 0.360577 | 0.331596 |
| 4.58043 | 0.630202 | 0.465745 | 0.284225 |
| 4.63006 | 0.535077 | 0.480769 | 0.473709 |
| 4.68022 | 0.58264 | 0.585938 | 0.473709 |
| 4.73093 | 0.558859 | 0.420673 | 0.355282 |
| 4.78219 | 0.558859 | 0.375601 | 0.568451 |
| 4.834 | 0.463734 | 0.555889 | 0.497395 |
| 4.88637 | 0.487515 | 0.465745 | 0.307911 |
| 4.93932 | 0.511296 | 0.465745 | 0.402653 |
| 4.99283 | 0.487515 | 0.495793 | 0.52108 |
| 5.04693 | 0.404281 | 0.360577 | 0.639507 |
| 5.10161 | 0.380499 | 0.315505 | 0.592136 |
| 5.15688 | 0.356718 | 0.450721 | 0.213169 |
| 5.21275 | 0.416171 | 0.315505 | 0.615822 |
| 5.26923 | 0.463734 | 0.360577 | 0.592136 |
| 5.32632 | 0.511296 | 0.405649 | 0.450024 |
| 5.38403 | 0.439952 | 0.435697 | 0.355282 |
| 5.44236 | 0.499405 | 0.450721 | 0.426338 |
| 5.50133 | 0.344828 | 0.390625 | 0.355282 |
| 5.56093 | 0.416171 | 0.435697 | 0.52108 |
| 5.62118 | 0.380499 | 0.390625 | 0.450024 |
| 5.68208 | 0.368609 | 0.285457 | 0.378967 |
| 5.74364 | 0.321046 | 0.420673 | 0.426338 |
| 5.80587 | 0.273484 | 0.270433 | 0.402653 |
| 5.86878 | 0.344828 | 0.330529 | 0.236855 |
| 5.93236 | 0.404281 | 0.210337 | 0.307911 |
| 5.99664 | 0.321046 | 0.360577 | 0.355282 |
| 6.06161 | 0.285375 | 0.375601 | 0.615822 |
| 6.12728 | 0.166468 | 0.315505 | 0.331596 |
| 6.19367 | 0.309156 | 0.180288 | 0.378967 |
| 6.26077 | 0.273484 | 0.180288 | 0.236855 |
| 6.3286 | 0.225922 | 0.375601 | 0.544766 |
| 6.39717 | 0.237812 | 0.240385 | 0.284225 |
| 6.46648 | 0.321046 | 0.195313 | 0.307911 |
| 6.53654 | 0.142687 | 0.240385 | 0.378967 |
| 6.60736 | 0.237812 | 0.225361 | 0.26054 |
| 6.67895 | 0.154578 | 0.195313 | 0.307911 |
| 6.75131 | 0.225922 | 0.255409 | 0.284225 |
| 6.82446 | 0.130797 | 0.15024 | 0.26054 |
| 6.8984 | 0.19025 | 0.225361 | 0.284225 |
| 6.97314 | 0.142687 | 0.120192 | 0.26054 |
| 7.04869 | 0.214031 | 0.210337 | 0.189484 |
| 7.12506 | 0.19025 | 0.180288 | 0.213169 |
| 7.20225 | 0.178359 | 0.15024 | 0.284225 |
| 7.28029 | 0.154578 | 0.0751202 | 0.236855 |
| 7.35917 | 0.107015 | 0.240385 | 0.213169 |
| 7.4389 | 0.142687 | 0.0901442 | 0.284225 |
| 7.5195 | 0.154578 | 0.210337 | 0.189484 |
| 7.60097 | 0.178359 | 0.120192 | 0.142113 |
| 7.68332 | 0.166468 | 0.0751202 | 0.307911 |
| 7.76656 | 0.0951248 | 0.180288 | 0.165798 |
| 7.85071 | 0.107015 | 0.105168 | 0.142113 |
| 7.93577 | 0.107015 | 0.0751202 | 0.142113 |
| 8.02175 | 0.0237812 | 0.135216 | 0.0947418 |
| 8.10866 | 0.0475624 | 0.225361 | 0.165798 |
| 8.19651 | 0.0832342 | 0.120192 | 0.142113 |
| 8.28532 | 0.142687 | 0.135216 | 0.189484 |
| 8.37508 | 0.059453 | 0.0300481 | 0.118427 |
| 8.46582 | 0.0475624 | 0.135216 | 0.165798 |
| 8.55755 | 0.059453 | 0.0751202 | 0.0473709 |
| 8.65026 | 0.0832342 | 0.0450721 | 0.142113 |
| 8.74398 | 0.107015 | 0.0450721 | 0.0947418 |
| 8.83872 | 0.0832342 | 0.120192 | 0.0710564 |
| 8.93448 | 0.0832342 | 0.105168 | 0.142113 |
| 9.03128 | 0.107015 | 0.0450721 | 0.142113 |
| 9.12913 | 0.0951248 | 0.0450721 | 0.118427 |
| 9.22804 | 0.0118906 | 0.015024 | 0.189484 |
| 9.32802 | 0.0713436 | 0.105168 | 0.0473709 |
| 9.42909 | 0.0475624 | 0.0450721 | 0.0710564 |
| 9.53125 | 0.0118906 | 0.0751202 | 0.0473709 |
| 9.63451 | 0.0713436 | 0.0300481 | 0.0473709 |
| 9.7389 | 0.059453 | 0.0450721 | 0.0236855 |
| 9.84441 | 0.118906 | 0.0450721 | 0.165798 |
| 9.95107 | 0.059453 | 0.0901442 | 0.0473709 |
| 10.0589 | 0.0118906 | 0.015024 | 0.0710564 |
| 10.1679 | 0.0356718 | 0.0450721 | 0.0710564 |
| 10.278 | 0.0356718 | 0.0450721 | 0.0236855 |
| 10.3894 | 0.0475624 | 0.0751202 | 0.0947418 |
| 10.502 | 0.0237812 | 0.0300481 | 0.0236855 |
| 10.6157 | 0 | 0.0450721 | 0.0947418 |
| 10.7308 | 0.0237812 | 0 | 0.142113 |
| 10.847 | 0.0356718 | 0.0300481 | 0.0947418 |
| 10.9645 | 0.0237812 | 0.0450721 | 0.0236855 |
| 11.0833 | 0.0118906 | 0.0751202 | 0.0236855 |
| 11.2034 | 0.0356718 | 0.015024 | 0.0236855 |
| 11.3248 | 0.0475624 | 0 | 0.0947418 |
| 11.4475 | 0.0118906 | 0.0450721 | 0.0236855 |
| 11.5715 | 0.0237812 | 0.0450721 | 0 |
| 11.6969 | 0.0237812 | 0.0450721 | 0.0473709 |
| 11.8236 | 0.0237812 | 0.0300481 | 0.142113 |
| 11.9517 | 0.0475624 | 0.0450721 | 0 |
| 12.0812 | 0.0237812 | 0.0450721 | 0.0236855 |
| 12.2121 | 0.0475624 | 0.0600962 | 0 |
| 12.3444 | 0.0237812 | 0.0300481 | 0.0236855 |
| 12.4782 | 0.0118906 | 0.0600962 | 0.0473709 |
| 12.6134 | 0.0356718 | 0 | 0 |
| 12.75 | 0 | 0.015024 | 0 |
| 12.8882 | 0.0118906 | 0 | 0 |
| 13.0278 | 0.0118906 | 0.015024 | 0.0236855 |
| 13.1689 | 0.0237812 | 0.015024 | 0.0236855 |
| 13.3116 | 0.0118906 | 0 | 0.0473709 |
| 13.4559 | 0 | 0 | 0.0236855 |
| 13.6016 | 0 | 0.015024 | 0.0473709 |
| 13.749 | 0 | 0 | 0.0473709 |
| 13.898 | 0.0237812 | 0.0300481 | 0 |
| 14.0485 | 0.0118906 | 0.0450721 | 0.0236855 |
| 14.2008 | 0 | 0.015024 | 0 |
| 14.3546 | 0 | 0.015024 | 0.0236855 |
| 14.5101 | 0 | 0 | 0.0236855 |
| 14.6673 | 0.0118906 | 0.015024 | 0 |
| 14.8263 | 0 | 0.0300481 | 0.0473709 |
| 14.9869 | 0.0118906 | 0.015024 | 0.0473709 |
| 15.1493 | 0 | 0.015024 | 0.0473709 |
| 15.3134 | 0.0237812 | 0.015024 | 0 |
| 15.4793 | 0 | 0.015024 | 0 |
| 15.647 | 0.0237812 | 0.015024 | 0 |
| 15.8166 | 0 | 0.015024 | 0 |
| 15.9879 | 0 | 0.015024 | 0 |
| 16.1611 | 0.0118906 | 0 | 0.0236855 |
| 16.3362 | 0.0237812 | 0 | 0 |
| 16.5132 | 0 | 0 | 0.0473709 |
| 16.6921 | 0 | 0 | 0 |
| 16.873 | 0 | 0.0300481 | 0.0236855 |
| 17.0558 | 0 | 0 | 0.0236855 |
| 17.2406 | 0 | 0 | 0 |
| 17.4274 | 0 | 0.015024 | 0.0473709 |
| 17.6162 | 0 | 0.015024 | 0 |
| 17.8071 | 0 | 0 | 0 |

**Supplementary Table S2.** Oxygen % saturation in vitro before and after 300 μL OMB (n=3 independent samples) or NMB (n=3 independent samples) injection intro into 70 mL partially degassed water

|  | **Baseline** | **Maximum reached over 5min** |
| --- | --- | --- |
| OMB trial 1 | 78.9 | 88.5 |
| OMB trial 2 | 80.3 | 102.8 |
| OMB trial 3 | 82.8 | 93.4 |
| NMB trial 1 | 79.8 | 80.8 |
| NMB trial 2 | 83.4 | 85.1 |
| NMB trial 3 | 86.0 | 89.0 |

**Supplementary Table S3.** Tumoral % hemoglobin saturation in a rat FSA model before and after OMB (n=4) or NMB (n=4) administration

|  | **Baseline** | **Peak change post injection** |
| --- | --- | --- |
| OMB animal 1 | 21.1 | 62.5 |
| OMB animal 2 | 50.8 | 100.0 |
| OMB animal 3 | 72.6 | 79.2 |
| OMB animal 4 | 67.5 | 82.6 |
| NMB animal 1 | 0 | 0 |
| NMB animal 2 | 83.4 | 81.8 |
| NMB animal 3 | 76.5 | 63.4 |
| NMB animal 4 | 69.5 | 49.3 |

**Supplementary Table S4.** Radiotherapy experiments initial tumor volumes for treatment group matching and stratification

|  | **No Treatment** | **RT** | **RT+NMB** | **RT+OMB** | **OMB alone** |
| --- | --- | --- | --- | --- | --- |
| initial tumor volume for each animal (cm^3^) | 0.11 | 0.10 | 0.13 | 0.10 | 0.08 |
|  | 0.22 | 0.29 | 0.28 | 0.45 | 0.29 |
|  | 0.58 | ^a^ | 0.70 | 0.58 | 0.64 |
|  | 0.77 | 0.90 | 0.86 | 1.02 | 0.73 |
|  |  | 1.74 | 1.86 | 1.52 |  |
|  |  | 3.35 | 2.69 | 2.59 |  |

^a^ Animal died prior to experimentation end

**Supplementary Table S5.** Radiotherapy experiments tumor control time (time for tumor to reach 2.5 cm in any direction, in days)

| **Tumor control time, in days, for each animal** | **No treatment (n=4)** | **OMB alone (n=4)** | **RT + NMB**  **(n=6)** | **RT**  **(n=6)** | **RT + OMB**  **(n=6)** |
| --- | --- | --- | --- | --- | --- |
| Round 1 | 32 | 32 | 32 | 32 | 32 |
|  | 25 | 19 | 32 | 32 | 32 |
|  | 10 | 19 | 22 | ^a^ | 31 |
|  | 10 | 10 | 22 | 22 | 28 |
| Round 2 |  |  | 10 | 16 | 22 |
|  |  |  | 7 | 7 | 10 |

^a^ Animal died prior to experimentation end
